# Letter to the Editor: A Rare Case of Upper Gastrointestinal Bleeding due to Amyloidosis

**DOI:** 10.5152/tjg.2025.24476

**Published:** 2025-05-20

**Authors:** Ali Karataş, Halit Kandemir, Gülden Bilican, Mehmet Cindoruk, Tarkan Karakan

**Affiliations:** Department of Gastroenterology, Gazi University School of Medicine, Ankara, Türkiye

Dear Editor,

A unique case highlighting the relationship between gastrointestinal (GI) amyloidosis and upper GI bleeding is being presented by the authors. This case provides valuable insight into a rare clinical presentation and underlines the importance of considering amyloidosis in patients with unexplained GI bleeding.

A 65-year-old female patient presented with nausea, abdominal pain, and hematemesis. She reported a significant weight loss of 20 kg over the past 6 months. Her medical history included sleeve gastrectomy 7 years ago, hypertension, and asthma but no prior history of GI bleeding. Vital signs on admission included a blood pressure of 110/70 mmHg, a heart rate of 90 beats/min, and an oxygen saturation of 95%. Physical examination revealed petechiae on the oral mucosa, pericardial friction rub, and bilateral lung rhonchi. Initial laboratory findings showed a hemoglobin level of 9.3 g/dL (baseline 12.0 g/dL) (range: 12-14), mean corpuscular volume of 72.6 fL (range: 80-100), ferritin of 7.8 ng/mL (range: 30-200), albumin of 3.1 g/dL (range: 4-4.8), and total protein of 8.1 g/dL (range: 6-8). Platelet count, prothrombin time, and tumor markers were within normal limits. Abnormal platelet function tests were noted due to the petechiae. Upper GI endoscopy revealed a hemorrhagic and fragile mucosal irregularity in a 4-5 cm segment in the second part of the duodenum (Figure 1). Active mucosal bleeding was controlled using “*Ankaferd Blood* Stopper®” and biopsy samples were taken for histopathological evaluation. Considering the patient’s general condition was not very good and the inability to tolerate repeated procedures, a biopsy was taken from the lesion during the first endoscopy session. Additionally, a second-look endoscopy was not performed due to the absence of a hemoglobin decrease, melena, and based on the patient’s overall condition. The patient was started on proton pump inhibitor therapy, and follow-up endoscopy showed no recurrent bleeding. Written informed consent was obtained from the patient.

Duodenal biopsy results showed positive staining consistent with amyloid deposition predominantly in the lamina propria, detected using crystal violet and Congo red staining (Figure 2). Immunohistochemistry was positive for Amyloid A and negative for Kappa and Lambda light chains, consistent with secondary (AA) amyloidosis. Considering anemia, bleeding diathesis, effusion, and a decrease in the albumin/globulin ratio, all of which raised clinical suspicion, multiple myeloma was strongly suspected and further investigated.

Further investigations revealed serum Kappa light chain levels of 287 mg/L, Lambda light chain levels of 15.1 mg/L, and a Kappa/Lambda ratio of 19. Upon the request of hematologists, a myocardial biopsy was planned since the treatment protocol would change according to the subtype of amyloidosis. The myocardial biopsy result was reported as AL (Amyloid Light Chain) amyloidosis (Figure 2). Hypercellular bone marrow with kappa monotypic plasma cell infiltration identified in the bone marrow biopsy, along with myocardial biopsy findings consistent with AL-type amyloidosis, led to the diagnosis of multiple myeloma, and chemotherapy was planned for the patient.

Upper GI tract hemorrhages originate from the proximal segment of the Treitz ligament, with the most common causes being peptic ulcer disease, hemorrhages related to esophagitis, gastritis, duodenitis, and variceal bleeding.^1^ Amyloidosis, characterized by extracellular deposition of misfolded fibrillar proteins, is a rare etiology for GI bleeding. The pathophysiology of GI bleeding from amyloidosis includes local ischemia, infarction, and mucosal damage causing erosions, hematomas, or ulcerations. It can be classified into primary (AL) and secondary (AA) forms based on the amyloidogenic precursor protein. While AL amyloidosis is often associated with plasma cell dyscrasias like multiple myeloma, AA amyloidosis typically results from chronic inflammatory diseases.^2^

Localized GI amyloidosis, as described in the systematic review by Malone et al., was analyzed in 62 cases, and it most commonly affected the stomach. Abdominal pain was the most frequent presenting symptom, followed by others such as GI bleeding, weight loss, and diarrhea. While the condition primarily involves the stomach, other sites, including the small and large intestines, can also be affected, with the jejunum and duodenum being the most common small intestinal locations. The review noted a higher prevalence in males and a median age of diagnosis in the mid-sixties.^3^ These findings highlight the varied anatomical involvement and clinical presentations, emphasizing the diagnostic challenges posed by this rare condition.

Our case highlights a rare presentation of AA amyloidosis in a duodenal biopsy alongside multiple myeloma, with AL amyloid deposits identified in myocardial tissue. Interestingly, in our patient, while the biopsy result from the duodenum indicated AA amyloidosis, the myocardial biopsy revealed AL amyloidosis associated with myeloma. Following a multidisciplinary evaluation by hematologists, pathologists, and gastroenterologists, it was concluded that our case involved concurrent AL amyloidosis due to myeloma and AA amyloidosis secondary to different causes, arising from 2 different clonalities. Similarly, Rosado et al. described a patient presenting with gastrointestinal bleeding, whose colonic biopsy revealed AA amyloidosis, ultimately leading to a diagnosis of multiple myeloma, emphasizing the unusual coexistence of AA amyloidosis with monoclonal gammopathy.^4^ Hu et al. also reported a case of multiple myeloma with systemic AL amyloidosis presenting initially with gastrointestinal symptoms such as abdominal pain and hematochezia.^5^ Upper gastrointestinal bleeding due to amyloidosis is considered a challenging condition to manage due to mucosal fragility and abnormal protein deposition in the vessel walls. In such cases, in addition to endoscopic treatment, alternative hemostatic agents have also been considered. The hemostatic effect of “*Ankaferd Blood* Stopper®” has been discussed in non-variceal upper gastrointestinal bleeding and has been proposed as a potential therapeutic option.^6^

These cases collectively underscore the diagnostic complexity of amyloidosis in patients with gastrointestinal bleeding, highlighting the importance of thorough histopathological evaluation, amyloid typing, and systemic assessment to guide proper diagnosis and management.

## Figures and Tables

**Figure 1.**
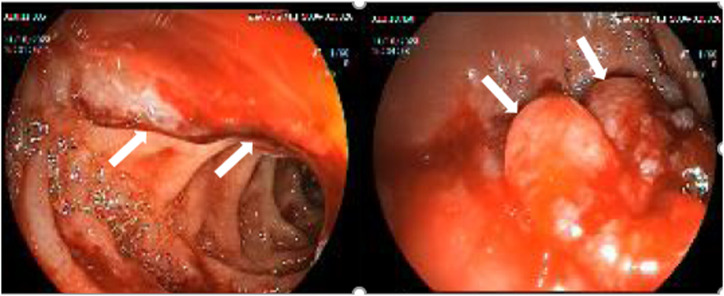
The area of fragile mucosa in part II of the duodenum: The irregular area rising from the mucosa is indicated by the white arrow.

**Figure 2.**
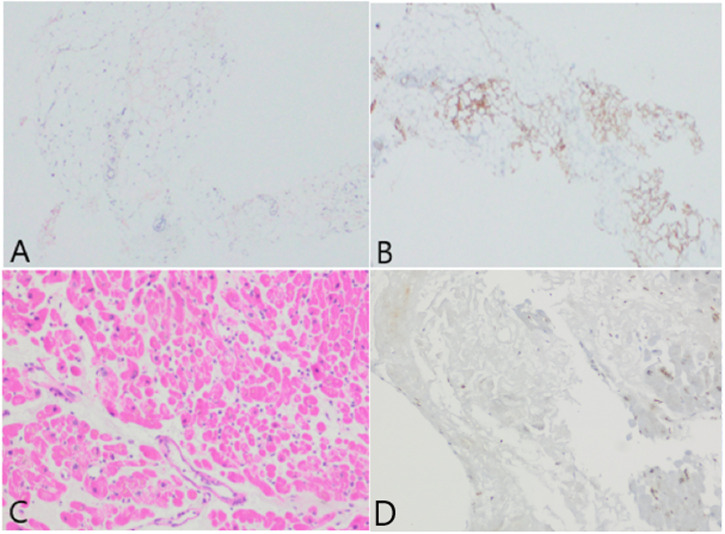
In the duodenal biopsy, widespread, weakly eosinophilic, amorphous Congo red-positive amyloid deposits were observed in both interstitial areas and vascular walls (A). In the immunohistochemical study, Amyloid A was noted to be diffusely and strongly positive in all deposition areas (B). In the myocardial biopsy, an amorphous appearance is observed in the extracellular area with hematoxylin-eosin staining (C). In the immunohistochemical study conducted on the areas of amyloid deposition, no staining with Amyloid A was observed (D).

## Data Availability

The data that support the findings of this study are available on request from the corresponding author.
